# Case Report: “Smart Palliation” and “Clepsydra Shape”: A new approach in complex congenital heart disease

**DOI:** 10.3389/fped.2022.1073412

**Published:** 2023-01-06

**Authors:** Ermanno Bellanti, Rita E. Calaciura, Ines Andriani, Michele Saitta, Salvatore Agati

**Affiliations:** Department of Congenital Heart Surgery and Pediatric Cardiology Mediterranean Congenital Heart Center, “ Bambino Gesù”—San Vincenzo Hospital, Taormina, Italy

**Keywords:** pediatric cardiosurgery, pediatric cardiologist, shunt, palliation surgery, innovation cardiologist

## Abstract

A limiting factor in using vascular conduits in the pediatric/newborn population is their inability to grow. Many complex congenital heart diseases require palliative surgery, but using rigid and nonexpandable conduits does not allow the structures to grow and anticipates the need for redo surgery. In newborns, a way to increase the palliation time according to the patient's growth is desirable. In recent years, expandable shunts (*exGraft™ PECA*) have been developed. According to recent material studies, a shunt could increase diameter after endovascular balloon dilatation. In this case report, we describe the first case of endovascular Blalock-Thomas-Taussig shunt (mBT) shunt expansion in a Tetralogy of Fallot / atrial-ventricular Septal Defect complete (TOFAVSDc) patient with trisomy 21 who went to palliative treatment for tracheomalacia (noncardiac lesion association), severe pulmonary arteries hypoplasia, and low weight. This case introduces the “*Smart Palliation concept*” in the clinical scenario of selected growing patients where the lifetime of the Blalock-Thomas-Taussig (BT) shunt, anatomic substrates, and complexity of clinical status may require an additional palliation time. The limitation of endovascular conduit expansion is the fragility of the anastomosis site. The anastomosis site is a lesser strength structure of the conduit, and dilatation could develop procedure complications. For this reason, in this paper, we introduced our project design: a new technique (*Clepsydra Shape*) that consists, before surgical implantation, of pre-expansion of the proximal and distal anastomotic parts of the shunt to obtain an increase of 30% in size of both anastomotic sides, preventing stress- and stretch-related lesion of future balloon dilatation.

## Introduction

**Figure 1:**
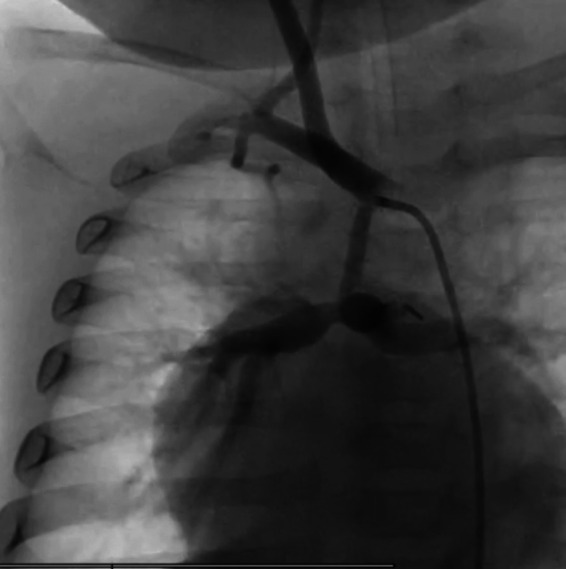
Angiography evidence of the restrictive flow pattern and the reduction size of the MRBT shunt (PECA exGraft).

**Figure 2:**
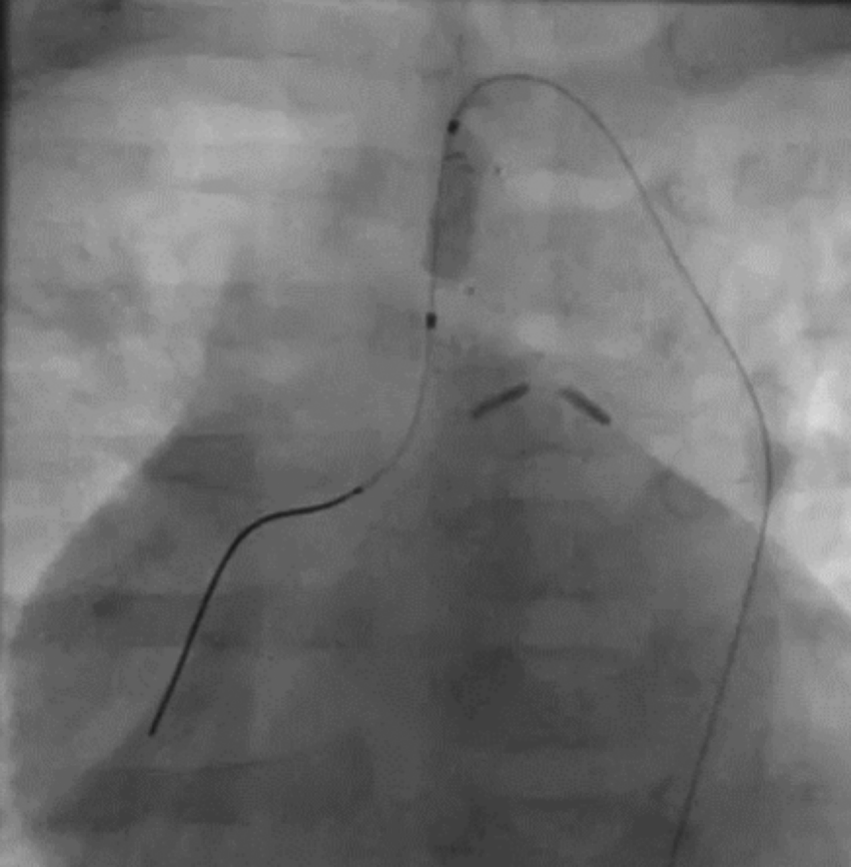
Intraprocedural frame of the mini-Tyshak balloon (NuMED For Children, Orlando, FL, United States) and MRBT shunt radiopaque reference alignment.

**Figure 3:**
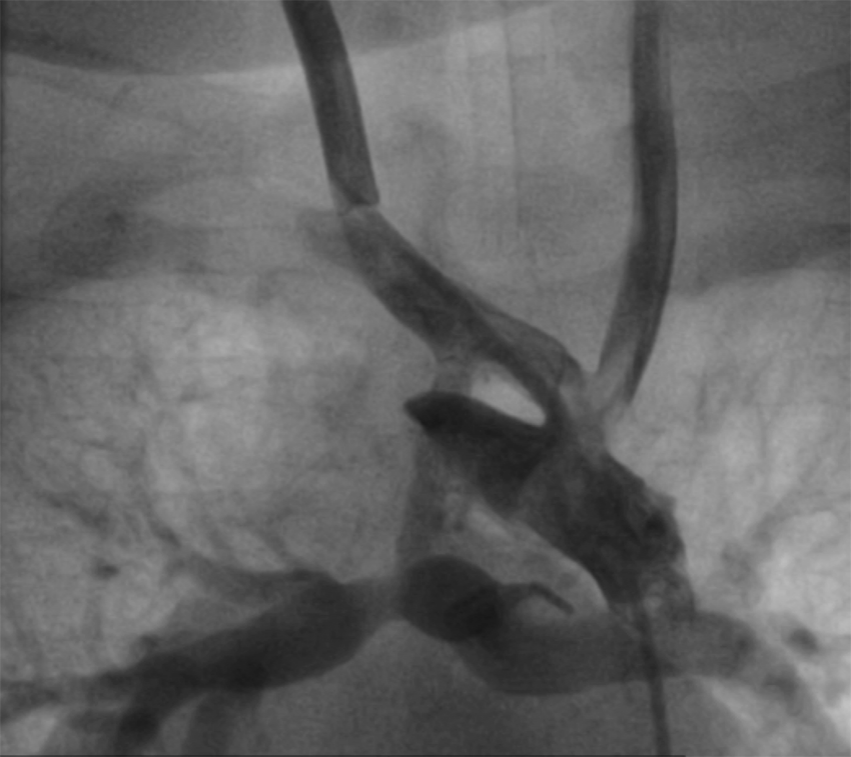
Hypoplastic diffusion BT shunt (2 mm) without significant stenosis. After guide placement, multiple dilations with 3/3.5/4 mm balloons are performed. The control BT shunt size was 4 mm.

Using conduits is common in the treatment of congenital heart diseases. Recently, the PECA exGraft™ shunt landed in clinical practice offering a percutaneous expandable option, which in selected cases could be used as a customized palliation strategy. Qadir et al. (1), in a single-ventricle disease neonate patient, described using a 6-mm PECA exGraft on the right ventricle–pulmonary artery conduit (Sano shunt). Instead, we report the first human case of PECA exGraft balloon dilatation in a newborn receiving an Blalock-Thomas-Taussig shunt (mBT) shunt for complex congenital heart disease. A 2.5-kg girl (trisomy 21) was referred to Mediterranean Congenital Heart Center with a diagnosis of Tetralogy of Fallot / atrial-ventricular Septal Defect complete (TOFAVSDc), severe pulmonary artery hypoplasia (Z score: 4.24), and subglottic stenosis in the tracheomalacia context. After 6 days of life, she underwent a 3.0-mm mBT shunt implantation without cardiopulmonary bypass; the postoperative period was free of complications. At 6 months of age, due to the saturation drop value of up to 75%, the patient was admitted to our center. Echocardiographic examination showed good biventricular function, patent but restrictive BT shunt flow pattern, and evidence of small pulmonary arteries. Cardiac catheterization was subsequently planned (Figure 1). Vascular access was taken on the right femoral artery: due to restriction of the PECA exGraft BT shunt (Figure 2), multiple dilatations were performed by mini-Tyshak (*NuMED For Children*, Orlando, FL, United States), raising from 3 mm to up to 4 mm × 20 mm balloon. The control shunt size was 4 mm in diameter, with a 33% increase in size (Figure 3). No signs of dissection or proximal and distal anastomosis distortion were detected. In our experience, “step-by-step dilatation” was preferred to reduce complications (especially at the anastomotic level) without increasing eccentric intensity in a short time, reducing the microstructural tension of the shunt. Our experience confirms the mechanical and structural parameters reported by *Loneker* et al. analyses (2). The patient was successfully extubated during the first postoperative day.

## Comment

A combination of tetralogy of Fallot and cAVSD is a well-recognized CHD that occurs in 5%–10% of patients with AVSD and 1.7% of patients with TOF (3). Controversies still exist about the timing of complete repair and the different surgical strategies (4). A palliative treatment is a well-accepted strategy in cyanotic patients or additional noncardiac lesions,. The choice of palliative treatment is influenced by various factors including age, low weight, clinical presentation severity, size of pulmonary arteries, tracheomalacia, and center preference. Due to the lack of clinical experience with PECA exGraft, we decide to start a clinical evaluation focusing on short and midterm results in the neonatal population. Since March 2020, 21 patients (mean shunt size: 3.59 mm, mean weight: 3.89 kg, and mean age: 28.63 days) received PECA exGraft shunts as palliation for complex CHD. A prospective analysis confirmed the efficacy and safety of the PECA exGraft shunt without complication or mortality rate. During our experience, due to the mechanical properties of the PECA exGraft™ shunt (1, 2, 5) and patient adaptation to the dilatation strategy, we were able to delay definitive correction 6 months after the balloon procedure without interstage-related complications and maintain hemodynamic stability. We suppose that type of syndrome, low birth weight, hypoplasia of the pulmonary arteries, and respiratory comorbidity were additional risk factors for one stage of complete surgical repair. This case introduces the *Smart Palliation concept* in the clinical scenario of selected growing patients where the lifetime of the BT shunt, anatomic substrates, and complexity of clinical status may require an additional palliation time. This approach may also be helpful in the adaptation of continuous pathophysiology changes directly related to congenital cardiac disease. This can reduce, delay, and personalize cardiac surgery procedures associated with high morbidity and mortality. Regarding the dilation procedure, several critical points have been advanced. It is well known that the distal and proximal anastomotic sites are the “*Achilles heels*” of percutaneous ballooning. Anastomotic shunt fragility and complication have already been reported in the literature (6). For this reason, the authors of this article are planning a method that could reduce this anastomosis tension and improve safety in endovascular dilation treatment.

**Figure 4:**
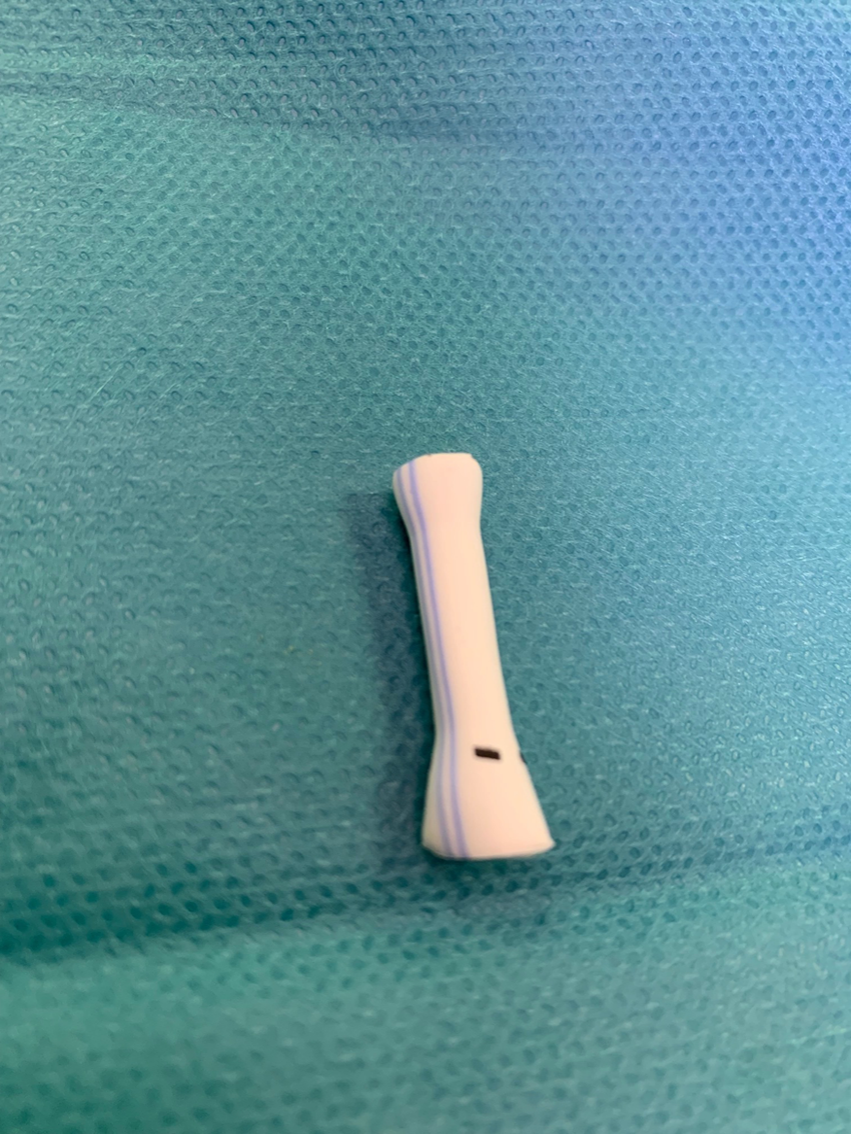
Predilatation view: distal and proximal parts of the future anastomotic site.

We are drawing and designing a new technique that consists, before surgical implantation, in pre-expansion (*Advance Low-Profile PTA Balloon Catheter—Cook Medical Europe Ltd.*) of the proximal and distal anastomotic parts of the shunt to obtain an increase of 30% in size of both anastomotic sides (Figure 4). Preflaring*—*called by us “*Clepsydra Shape*”*—*will respect the necessary pulmonary flow, preventing balloon stress- and stretch-related lesions of the suture line on the proximal and distal anastomosis, fixing the final conduit diameter. Shunt dilatation with a “step-by-step” balloon system was performed for the best dynamic control, increasing the proximal and distal diameters and dosing the concentric distribution forces. The Clepsydra Shape project is in the design phase, but the preclinical results are very encouraging and will be proposed for publication when available. With PECA BT shunt endovascular dilatation, we are able to decide the right dilation or complete surgical correction performing timing. This methodology makes it possible to rethink indications for palliative surgery. The anastomotic implantation technique is no different from that used with other shunt devices. Our center does not use a different anticoagulation protocol, but we are analyzing new clinical and histological dates that, in the future, could give new indications and directions. In our center, the PECA exGraft™ shunt has become the first choice in neonatal patients, where a small diameter increase allows a longer palliative window time. Extending palliation time is an “arrow in our quiver” that can be used according to the different clinical cases. This will allow us to perform the correction surgery with an adequate weight and pulmonary artery Z score increase. Our research group is working to develop a specific registry that could help understand the clinical impact of the Smart Palliation approach.

## Data Availability

The original contributions presented in the study are included in the article/Supplementary Material, further inquiries can be directed to the corresponding author.
